# Supplementary material for: Analysis of PPARγ Signaling Activity in Psoriasis
Source: Int J Mol Sci. 2021 Aug 10;22(16):8603. doi: 10.3390/ijms22168603 (PMC8395241; doi:10.3390/ijms22168603)
Supplement: Supplementary file 1 [file ijms-22-08603-s001.zip › Supplemental materials_Analysis of PPARg signaling activity in psoriasis/Pathway models/Models images and html files/Differentiation of psoriatic T cells/7707.html]

TGFBR1


# Protein TGFBR1

|  |  |
| --- | --- |
| URN | urn:agi-llid:4484 |
| Total Entities | 0 |
| Connectivity | 3496 |
| Name | TGFBR1 |
| Description | transforming growth factor beta receptor I |
| Notes | The protein encoded by this gene forms a heteromeric complex with type II TGF-beta receptors when bound to TGF-beta, transducing the TGF-beta signal from the cell surface to the cytoplasm. The encoded protein is a serine/threonine protein kinase. Mutations in this gene have been associated with Loeys-Dietz aortic aneurysm syndrome (LDAS). Multiple transcript variants encoding different isoforms have been found for this gene. [provided by RefSeq, Aug 2008] |
| Primary Cell Localization | Plasma membrane |
| Class | Receptor |

---

|  |  |
| --- | --- |
| Pathway | Osteoarthritis |
|  | Immunoglobulin Class-Switch Recombination Activation |
|  | Treg-Cell Differentiation |
|  | AHR Signaling in Treg and Dendritic Cells Function |
|  | AHR Signaling in Th17 Cells Function |
|  | Th17-Cell Differentiation |
|  | anti-Inflammatory Function of Macrophage M2 Lineage |
|  | Vascular Endothelial Cell Activation by Growth Factors |
|  | Vascular Smooth Muscle Cell/Pericyte Differentiation and Proliferation |
|  | PRL Expression Regulation by BMP4 and TGFB1 |
|  | G1/S Damage Checkpoint |
|  | Cell Cycle Overiew |
|  | Transcytosis |
|  | Cardiomyocyte Dysfunction |
|  | Dilated Cardiomyopathy |
|  | Cardiomyocyte Hypertrophy |
|  | Hypertrophic Cardiomyopathy |
|  | Proteins Involved in Pulmonary Hypertension |
|  | BMP/TGF-beta Signaling Impairment in Pulmonary Hypertension |
|  | Lymphocyte Mediated Myocardial Injury in Myocarditis |
|  | Hair Follicle Keratinocyte Apoptosis |
|  | Androgens in Sebocyte Maturation |
|  | Androgens Promote Scalp Dermal Papilla Regression |
|  | T-Cells Differentiation Block in Psoriasis |
|  | TGF-beta Signaling in Skin Aging |
|  | Treg-Cell Function in Diabetes Mellitus Type 1 |
|  | non-Suppressive Treg-Cell in Diabetes Mellitus Type 1 |
|  | TLR2 Signaling in Treg-Cell in Type 1 Diabetes (Animal Model) |
|  | Treg-Cell Activation in Diabetes Mellitus |
|  | Tubular Cell Dysfunction in Progressive Diabetic Nephropathy |
|  | Endothelial Cell Dysfunction in Progressive Diabetic Nephropathy |
|  | Proteins Involved in Diabetic Nephropathy |
|  | Mesangial Cell Dysfunction in Diabetic Nephropathy |
|  | Hepatic Stellate Cells in non-Alcoholic Fatty Liver Disease |
|  | Metastatic Colorectal Cancer |
|  | GUCY2C Signaling in Colorectal Cancer |
|  | Th17-Cell Activation in Crohn's Disease |
|  | Chronic Myeloid Leukemia |
|  | Proteins Involved in Hepatocellular Carcinoma |
|  | Hepatocellular Carcinoma |
|  | Growth Factor Signaling in Hepatocellular Carcinoma |
|  | Proteins Involved in Pancreatic Cancer |
|  | Pancreatic Ductal Carcinoma |
|  | Pancreatic Neuroendocrine Tumors |
|  | TGFBR Signaling in Pancreatic Neoplasms |
|  | ADAM33 Role in Asthma |
|  | Th17-Cell Differentiation in Asthma |
|  | Osteoblast Function Decline in Gout |
|  | TGFB2 Signaling Impairement in Osteoarthritis |
|  | Th17-Cell and Th1 Immune Responsein Psoriatic Arthritis |
|  | Autocrine Cytokine/Chemokine Loops in Systemic Scleroderma |
|  | Skin Fibrosis |
|  | Th17-Cell Numbers Reduction in HIV |
|  | Hereditary Hemorrhagic Telangiectasia |
|  | Myostatin in Skeletal Muscles in Muscular Dystrophies |
|  | Myostatin-IGF1 Crosstalk in Skeletal Muscles in Muscular Dystrophies |
|  | Proteins with Altered Expression in Osteoarthritis |
|  | Bone Loss in Osteoporosis |
|  | Hyperparathyroidism, Secondary Effect |
|  | Blood-Brain Barrier Disruption in Epileptiform Disorders |
|  | Glioma Stem Cell Program Activation |
|  | Medulloblastoma |
|  | Proteins with Altered Expression in Breast Cancer |
|  | Breast Cancer |
|  | Ovarian Cancer |
|  | Proteins with Altered Expression in Ovarian Cancer |
|  | BMP Signaling Impairment in Granulosa Cell in POF |
|  | Prostate Cancer |
|  | Proteins Involved in Prostate Cancer |
|  | Cytokines Trigger Otitis Media |
|  | Podocyte Dysfunction in Glomerulonephritis |
|  | Mesangial Cell Dysfunction in Glomerulonephritis |
|  | Interstitial Fibroblasts in Pyelonephritis |
|  | TGFBR3 -> ARRB2 Signaling |
|  | Dexamethasone Induced Osteoporosis |
|  | Proteins with Altered Expression in Cancer-Associated Sustaining of Proliferative Signaling |
|  | Genes with Mutations in Cancer-Associated Sustaining of Proliferative Signaling |
|  | DNA Damage Checkpoint Impairment in Cancer |
|  | IL23A and IL17A Provoke Cancer-Associated Inflammation |
|  | N2 Neutrophils in Tumor-Promoting Inflammation and Tumor Progression |
|  | TGFB Signaling activation by Blocking of Tumor Suppressors |
|  | Genes with Mutations in Cancer-Associated Evading of Growth Suppressors |
|  | Proteins with Altered Expression in Cancer-Associated Evading of Growth Suppressors |
|  | HIF1A in Vasculogenic Mimicry of Cancer |
|  | FBLN5 Potentiates Migration and Proliferation of Endothelial Cells in Cancer |
|  | Hyaluronic Acid, CD44 and HMMR in Cancer Cell Invasion and Survival |
|  | TGFB Family in Epithelial to Mesenchymal Transition in Cancer |
|  | Invadopodia Formation in Cancer Cells |
|  | Skeletal Muscle Wasting in Cancer Cachexia |
|  | Treg Cells Promote Immunosuppression in Cancer Immune Escape |
|  | TGFBR -> AP-1 Signaling |
|  | TGFBR/BMPR -> SMAD2/3 Signaling |
|  | TGFBR -> CREB/ELK-SRF Signaling |
|  | TGFBR -> ATF/GADD/MAX/TP53 Signaling |
|  | TGFBR -> MEF/MYOD/MYOG Signaling |
|  | TGFBR1/AP-1 Signaling |
|  | TGF-beta Signaling |
|  | TGFB2-TGFBR1 Expression Targets |
|  | TGFB3-TGFBR1 Expression Targets |
|  | TGFB1-TGFBR1 Expression Targets |
|  | TGFB1-TGFBR1/AP-1 Expression Targets |
|  | PPAR Psoriasis |
|  | prarg negative regulators, ps-positive |
|  | Model of PPARG signaling in psoriasis |
|  | PPARG negative regulators and targets |
|  | Model of PPARG related pathways in psoriasis (short version) |
|  | 1\_Differentiation of psoriatic T cells |
|  | 3\_TGFB signaling provokes endochondral ossification with osteophyte formation |
|  | 3\_The role of interstitial fibroblasts in pyelonephritis |
|  | 3\_Mesangial cell dysfunction in glomerulonephritis |
|  | 2\_Podocyte dysfunction in glomerulonephritis |
|  | 3\_Increased proliferation of pulmonary artery smooth muscle cells (PASMCs) in familial forms of pulmonary hypertension |
|  | 2\_Cardiomyocyte hypertrophy |
|  | 3\_Cardiomyocyte dysfunction unrelated to cell junctions in ARVD |
|  | 4\_2\_Genes with mutations currently associated with ulcerative colitis |
|  | 4\_1\_Polymorphisms associated with inflammatory bowel diseases |
|  | 2\_Defective tolerance of autoreactive T-cell in T1D |
|  | Differentiation of psoriatic T cells |

---

|  |  |
| --- | --- |
| Group | Genes with Susceptibility Loci Associated with Age-Related Macular Degeneration |
|  | Chemokines, Growth Factors and Receptors Involved in Cancer Cells Chemotaxis |

---

|  |  |
| --- | --- |
| MedScan ID | 4484 |

---

|  |  |
| --- | --- |
| LocusLink ID | 4484 |
|  | 7046 |
|  | 21812 |
|  | 29591 |

---

|  |  |
| --- | --- |
| Alias | T beta R-I |
|  | TGF-beta type I and activin receptors |
|  | Transforming Growth Factor-beta RI |
|  | OTTMUSP00000007207 |
|  | OTTHUMP00000021783 |
|  | AU017191 |
|  | activin A receptor type II-like kinase, 53kD |
|  | transforming growth factor beta (TGF-beta) receptor type I |
|  | TGF-beta R I |
|  | TGF-beta type I and activin receptor |
|  | TGF-beta 1 receptor |
|  | Tgfbr I |
|  | ESK2 |
|  | OTTMUSP00000007205 |
|  | activin A receptor type II-like kinase |
|  | ALK V |
|  | MSSE gene |
|  | TGFR-1 |
|  | TGF-beta 1 receptors |
|  | AAT5 |
|  | TbetaRI |
|  | TGF-beta I receptors |
|  | TGFR I |
|  | RP23-457P12.1 |
|  | transforming growth factor-beta serine/threonine type I receptors |
|  | TGF-beta type I receptor |
|  | type I transforming growth factor-beta receptor |
|  | ESS I |
|  | TGF-beta 1Rs |
|  | Transforming Growth Factor-beta R I |
|  | MGC93659 |
|  | activin A receptor type II-like kinase, 53kDa |
|  | Activin receptor-like kinase 5 |
|  | transforming growth factor beta receptor I |
|  | TGF-beta receptor type I |
|  | type I transforming growth factor-beta receptors |
|  | multiple self-healing squamous epithelioma gene |
|  | LDS2A |
|  | transforming growth factor beta 1 receptor |
|  | transforming growth factor beta 1 receptors |
|  | T beta RI |
|  | LOC666236 |
|  | ALK-5 |
|  | TGFb1R |
|  | TGF-beta RI |
|  | multiple self-healing squamous epithelioma protein |
|  | LOC669553 |
|  | TbetaR-I |
|  | ESS1 |
|  | SKR4 |
|  | LOC674605 |
|  | TGFBR1 |
|  | LDS1A |
|  | transforming growth factor-beta serine/threonine type I receptor |
|  | TGF-beta 1R |
|  | TGFb1Rs |
|  | TGR1\_HUMAN |
|  | RP11-96L7.1 |
|  | TGF-beta receptor type-1 |
|  | TGR I |
|  | TBRI |
|  | Serine/threonine-protein kinase receptor R4 |
|  | Activin receptor-like kinase V |
|  | transforming growth factor, beta receptor 1 |
|  | TGR1 |
|  | ALK5 |
|  | LDS1 |
|  | MSSE |
|  | TBR-i |
|  | ACVRLK4 |
|  | activin A receptor type II-like protein kinase of 53kD |
|  | mutant transforming growth factor beta receptor I |
|  | transforming growth factor-beta receptor type I |
|  | transforming growth factor, beta receptor I |
|  | transforming growth factor, beta receptor I (activin A receptor type II-like kinase, 53kD) |

---

|  |  |
| --- | --- |
| EC Number | 2.7.11.30 |

---

|  |  |
| --- | --- |
| GO ID | 0005524 |
|  | 0070411 |
|  | 0046332 |
|  | 0048185 |
|  | 0019838 |
|  | 0046872 |
|  | 0004672 |
|  | 0004674 |
|  | 0050431 |
|  | 0005025 |
|  | 0005024 |
|  | 0005114 |
|  | 0000186 |
|  | 0032924 |
|  | 0060978 |
|  | 0009952 |
|  | 0006915 |
|  | 0048844 |
|  | 0001824 |
|  | 0060317 |
|  | 0007050 |
|  | 0048870 |
|  | 0071560 |
|  | 0030199 |
|  | 0060982 |
|  | 0048701 |
|  | 0042118 |
|  | 0043542 |
|  | 1905223 |
|  | 0001837 |
|  | 0043062 |
|  | 0008354 |
|  | 0007507 |
|  | 0001701 |
|  | 0035556 |
|  | 0001822 |
|  | 0002088 |
|  | 0008584 |
|  | 0048762 |
|  | 0032331 |
|  | 0001937 |
|  | 2001237 |
|  | 0030512 |
|  | 0048663 |
|  | 0060017 |
|  | 0060389 |
|  | 0007389 |
|  | 0018105 |
|  | 0018107 |
|  | 0060037 |
|  | 0060391 |
|  | 2001235 |
|  | 0030307 |
|  | 0030335 |
|  | 0008284 |
|  | 0051272 |
|  | 0001938 |
|  | 0010718 |
|  | 1905007 |
|  | 0051491 |
|  | 0010628 |
|  | 0010862 |
|  | 0051897 |
|  | 0051496 |
|  | 1905075 |
|  | 0045893 |
|  | 0009791 |
|  | 0003342 |
|  | 0016579 |
|  | 0006468 |
|  | 0060043 |
|  | 0010717 |
|  | 0043393 |
|  | 0031396 |
|  | 0006355 |
|  | 0070723 |
|  | 0060021 |
|  | 0007165 |
|  | 0001501 |
|  | 0048705 |
|  | 0048538 |
|  | 0007179 |
|  | 0003223 |
|  | 0060412 |
|  | 0003222 |
|  | 0042060 |
|  | 0048179 |
|  | 0005923 |
|  | 0009986 |
|  | 0005768 |
|  | 0005887 |
|  | 0043231 |
|  | 0045121 |
|  | 0005634 |
|  | 0005886 |
|  | 0043235 |
|  | 0016301 |
|  | 0000166 |
|  | 0046982 |
|  | 0044877 |
|  | 0005102 |
|  | 0016740 |
|  | 0004675 |
|  | 0031625 |
|  | 0001525 |
|  | 0030154 |
|  | 0043066 |
|  | 0045602 |
|  | 1902894 |
|  | 0016310 |
|  | 0043065 |
|  | 1902895 |
|  | 0046777 |
|  | 0010468 |
|  | 0040008 |
|  | 0043627 |
|  | 0007178 |
|  | 0016324 |
|  | 0016323 |
|  | 0005901 |
|  | 0030054 |
|  | 0016021 |
|  | 0016020 |
|  | 0032991 |
|  | 0007568 |
|  | 0031100 |
|  | 0048565 |
|  | 0007566 |
|  | 0030324 |
|  | 0051602 |
|  | 0001666 |
|  | 0014070 |
|  | 0034695 |
|  | 0009636 |
|  | 0005623 |
|  | 0005622 |
|  | 0070022 |
|  | 0005057 |
|  | 0004702 |
|  | 0023014 |
|  | 0032403 |
|  | 0009790 |
|  | 0043234 |
|  | 0016772 |

---

|  |  |
| --- | --- |
| KEGG ID | hsa:7046 |
|  | mmu:21812 |

---

|  |  |
| --- | --- |
| Organism | Homo sapiens {Organism urn:agi-taxid:9606} |
|  | Mus musculus {Organism urn:agi-taxid:10090} |
|  | Rattus norvegicus {Organism urn:agi-taxid:10116} |
|  | Homo sapiens |
|  | Mus musculus |
|  | Rattus norvegicus |

---

|  |  |
| --- | --- |
| Mouse chromosome position | 4 26.02 cM |

---

|  |  |
| --- | --- |
| OMIM ID | 190181 |
|  | 609192 |
|  | 132800 |

---

|  |  |
| --- | --- |
| Rat chromosome position | 5q22 |

---

|  |  |
| --- | --- |
| Hugo ID | 11772 |
|  | HGNC:11772 |

---

|  |  |
| --- | --- |
| Human chromosome position | 9q22.33 |
|  | 9q22 |

---

|  |  |
| --- | --- |
| Swiss-Prot Accession | B4DXN7 |
|  | P36897 |
|  | Q5T7S2 |
|  | B4DY26 |
|  | P36897.1 |
|  | Q64729 |
|  | Q9D5H8 |
|  | Q4FJL1 |
|  | Q64729.1 |
|  | Q5M9H3 |
|  | P80204 |
|  | P80204.1 |
|  | Q6IR47 |
|  | Q706C0 |
|  | Q706C1 |
|  | A2AJN0 |

---

|  |  |
| --- | --- |
| PIR ID | A49432 |
|  | JC2061 |
|  | JC2062 |

---

|  |  |
| --- | --- |
| GenBank ID | NC\_000009 |
|  | XM\_011518949 |
|  | XP\_011517251 |
|  | XM\_017015063 |
|  | XP\_016870552 |
|  | NM\_001306210 |
|  | NP\_001293139 |
|  | NM\_004612 |
|  | NP\_004603 |
|  | NM\_001130916 |
|  | NP\_001124388 |
|  | XM\_011518950 |
|  | XP\_011517252 |
|  | XM\_024447658 |
|  | XP\_024303426 |
|  | XM\_011518948 |
|  | XP\_011517250 |
|  | NG\_007461 |
|  | AH006005 |
|  | AAC08998 |
|  | AH007196 |
|  | AAD02042 |
|  | AH015633 |
|  | ABD46753 |
|  | AL162427 |
|  | AY497473 |
|  | AAR32097 |
|  | CH471105 |
|  | EAW58905 |
|  | EAW58906 |
|  | GU143401 |
|  | ACZ58375 |
|  | HB448229 |
|  | CAZ91468 |
|  | U51139 |
|  | AJ420419 |
|  | AJ619019 |
|  | CAF02096 |
|  | AJ619020 |
|  | CAF02097 |
|  | AK002171 |
|  | AK129793 |
|  | AK302059 |
|  | BAG63449 |
|  | AK302234 |
|  | BAG63588 |
|  | AL117597 |
|  | BC038347 |
|  | BC056247 |
|  | BC071181 |
|  | AAH71181 |
|  | BX648917 |
|  | EF142853 |
|  | ABO93068 |
|  | EF142854 |
|  | ABO93069 |
|  | L11695 |
|  | AAA16073 |
|  | P36897 |
|  | NC\_000070 |
|  | NM\_009370 |
|  | NP\_033396 |
|  | NM\_001312868 |
|  | NP\_001299797 |
|  | XM\_017320103 |
|  | XP\_017175592 |
|  | NM\_001312869 |
|  | NP\_001299798 |
|  | AL772150 |
|  | AL772232 |
|  | CH466565 |
|  | EDL02353 |
|  | FB746001 |
|  | CAT04599 |
|  | AK007125 |
|  | BAB24865 |
|  | AK015337 |
|  | BAB29802 |
|  | AK153796 |
|  | AK154381 |
|  | BAE32548 |
|  | BC063260 |
|  | AAH63260 |
|  | BY200044 |
|  | CK637995 |
|  | CT010393 |
|  | CAJ18600 |
|  | D25540 |
|  | BAA05023 |
|  | D28526 |
|  | BAA05878 |
|  | Q64729 |
|  | NC\_005104 |
|  | XM\_006238030 |
|  | XP\_006238092 |
|  | NM\_012775 |
|  | NP\_036907 |
|  | AC\_000073 |
|  | AABR07048119 |
|  | AABR07048120 |
|  | AAHX01036783 |
|  | CH474056 |
|  | EDL78209 |
|  | U48401 |
|  | BC087035 |
|  | AAH87035 |
|  | BC091352 |
|  | CB804748 |
|  | CK844086 |
|  | L26110 |
|  | AAA83216 |
|  | P80204 |
|  | XM\_005252150 |
|  | XP\_005252207 |
|  | NC\_018920 |
|  | AF035662 |
|  | AF035663 |
|  | AF035664 |
|  | AF035665 |
|  | AF035666 |
|  | AF035667 |
|  | AF035668 |
|  | AF035669 |
|  | AF035670 |
|  | AF054590 |
|  | AF054591 |
|  | AF054592 |
|  | AF054593 |
|  | AF054595 |
|  | AF054597 |
|  | AF054598 |
|  | AMYH02021328 |
|  | AMYH02021329 |
|  | DQ383416 |
|  | DQ383417 |
|  | DQ383418 |
|  | DQ383419 |
|  | DQ383420 |
|  | DQ383421 |
|  | DQ383422 |
|  | DQ383423 |
|  | DQ383424 |
|  | XM\_006537756 |
|  | XP\_006537819 |
|  | XM\_006537757 |
|  | XP\_006537820 |
|  | AC\_000026 |
|  | AAHY01035599 |
|  | AAHY01035600 |
|  | XR\_592491 |
|  | AC\_000141 |
|  | ABBA01042903 |
|  | ABBA01042904 |

---

|  |  |
| --- | --- |
| Swiss-Prot ID | TGFR1\_HUMAN |
|  | TGFR1\_MOUSE |
|  | TGFR1\_RAT |

---

|  |  |
| --- | --- |
| Cell Localization | Cell membrane |
|  | Cell junction |
|  | Tight junction |
|  | Cell surface |
|  | Membrane raft |
|  | Cell junction, tight junction |

---

|  |  |
| --- | --- |
| Ensembl ID | ENSG00000106799 |
|  | ENSP00000364129.2 |
|  | ENST00000374990.6 |
|  | ENSP00000447297.1 |
|  | ENST00000552516.5 |
|  | ENSP00000364133.4 |
|  | ENST00000374994.9 |
|  | ENSP00000450052.1 |
|  | ENST00000550253.1 |
|  | ENSMUSG00000007613 |
|  | ENSMUSP00000048501.7 |
|  | ENSMUST00000044234.13 |
|  | ENSMUSP00000103353.2 |
|  | ENSMUST00000107725.2 |
|  | ENSMUSP00000007757.8 |
|  | ENSMUST00000007757.14 |
|  | ENSMUSP00000123761.1 |
|  | ENSMUST00000126171.8 |
|  | ENSRNOG00000007036 |
|  | ENSRNOP00000071347.1 |
|  | ENSRNOT00000081090.1 |
|  | ENST00000374994.8 |
|  | ENSP00000364129 |
|  | ENST00000374990 |
|  | ENSP00000364133 |
|  | ENST00000374994 |
|  | ENSP00000450052 |
|  | ENST00000550253 |
|  | ENSP00000447297 |
|  | ENST00000552516 |
|  | ENSMUSP00000007757 |
|  | ENSMUST00000007757 |
|  | ENSMUSP00000048501 |
|  | ENSMUST00000044234 |
|  | ENSRNOP00000071347 |
|  | ENSRNOT00000081090 |
|  | ENSRNOG00000050760 |

---

|  |  |
| --- | --- |
| MGI ID | MGI:98728 |

---

|  |  |
| --- | --- |
| RGD ID | 3852 |

---

|  |  |
| --- | --- |
| Unigene ID | Rn.44402 |
|  | Hs.494622 |
|  | Mm.197552 |

---

|  |  |
| --- | --- |
| Homologene ID | 3177 |

---

|  |  |
| --- | --- |
| Shape | Stick-vertex |

---
